# Toll-like Receptor-Dependent Negative Effects of Opioids: A Battle between Analgesia and Hyperalgesia

**DOI:** 10.3389/fimmu.2017.00642

**Published:** 2017-05-31

**Authors:** Masaud Shah, Sangdun Choi

**Affiliations:** ^1^Department of Molecular Science and Technology, Ajou University, Suwon, South Korea

**Keywords:** hyperalgesia, morphine, opioid, pain, toll-like receptor

## Abstract

Our understanding of the pathophysiology of the pathological pain and the pharmacology of analgesic treatments has progressed tremendously over the past two decades. Among the well-documented pro-algesic factors, glia and other toll-like receptors (TLRs)-expressing cells in the neuroimmune interface have been recognized for their role in the development of neuropathic pain and for compromising the analgesic effects of opioids. Here, we comprehensively review the molecular mechanisms of pain initiation and progression, the role of TLRs in these processes, and the molecular mechanisms of morphine and morphine-3-glucuronide in TLR-dependent central immune signaling. The data reviewed here suggest that, while targeting glia to treat neuropathic pain, both analgesic and analgesia-opposing effects of opioids must be considered by acknowledging their role in TLR-mediated signaling.

## Introduction

The interaction between the immunoprivileged nervous system and the immune system is not just limited to inflammatory conditions, as emphasized by studies reporting a two-way signaling between immunocompetent cells, including microglia, oligodendrocytes, astrocytes, and endothelial and neuronal cells present in the central nervous systems (CNS) and peripheral nervous systems (PNS) (1, 2). The interaction between immune and neuronal cells is crucial for the trauma-induced sensitization and pathophysiological changes that occur at the site of peripheral nerve injury. Physical or chemical incitements activate neuroimmune cells present at the site of injury, lead to the release of chemokines and cytokines, and enhance the neuroimmune response by enhanced expression of surface antigens on reacting cells (2).

Both the inflammatory and the neuropathic pain have been linked only to neuronal mechanisms until recently. However, advances in our understanding of the underlying pathophysiological mechanism and growing interest in the etiology of pain have highlighted the contribution of neuroimmune cells in the development and persistence of pain (3–5). Overproduction of cytokines and chemokines by reactive immune cells and expression of danger-associated molecular patterns (DAMPs)-recognizing receptors on neurons often enhance sensitivity to hyperalgesic stimuli and subsequently result in nociception, as reviewed previously (6).

The mechanism underlying the involvement of glia and other neuroimmune cells in nociception has been well documented. It has recently been reported that opioids, which are considered the gold standard for the treatment of pain, can also induce enhanced sensitization of neuronal and immune cells present in the neuroimmune interface and thereby lead to paradoxical hyperalgesia. The molecular mechanism of pain and its progression has been well elaborated previously; however, here, we will briefly review the role played by toll-like receptor (TLR)-expressing immune cells in hyperalgesia, the molecular mechanism of TLR-mediated signaling in pain sensitization, and the involvement of morphine and morphine-3-glucuronide (M3G) in hyperalgesia with respect to TLR-mediated signaling.

## Neuroimmune Cells and Their Role in Pain Sensation

Glia, previously known to provide structural support to neuronal cells, is now recognized as playing a crucial role in the neuroimmune system, particularly in clearing cellular debris and providing immune surveillance (7). Nonetheless, glia is also endowed with the capacity to modulate pain and play a major role in sex-dependent pain sensitivity and the opioid response, as reviewed previously (7, 8).

**Figure 1:**
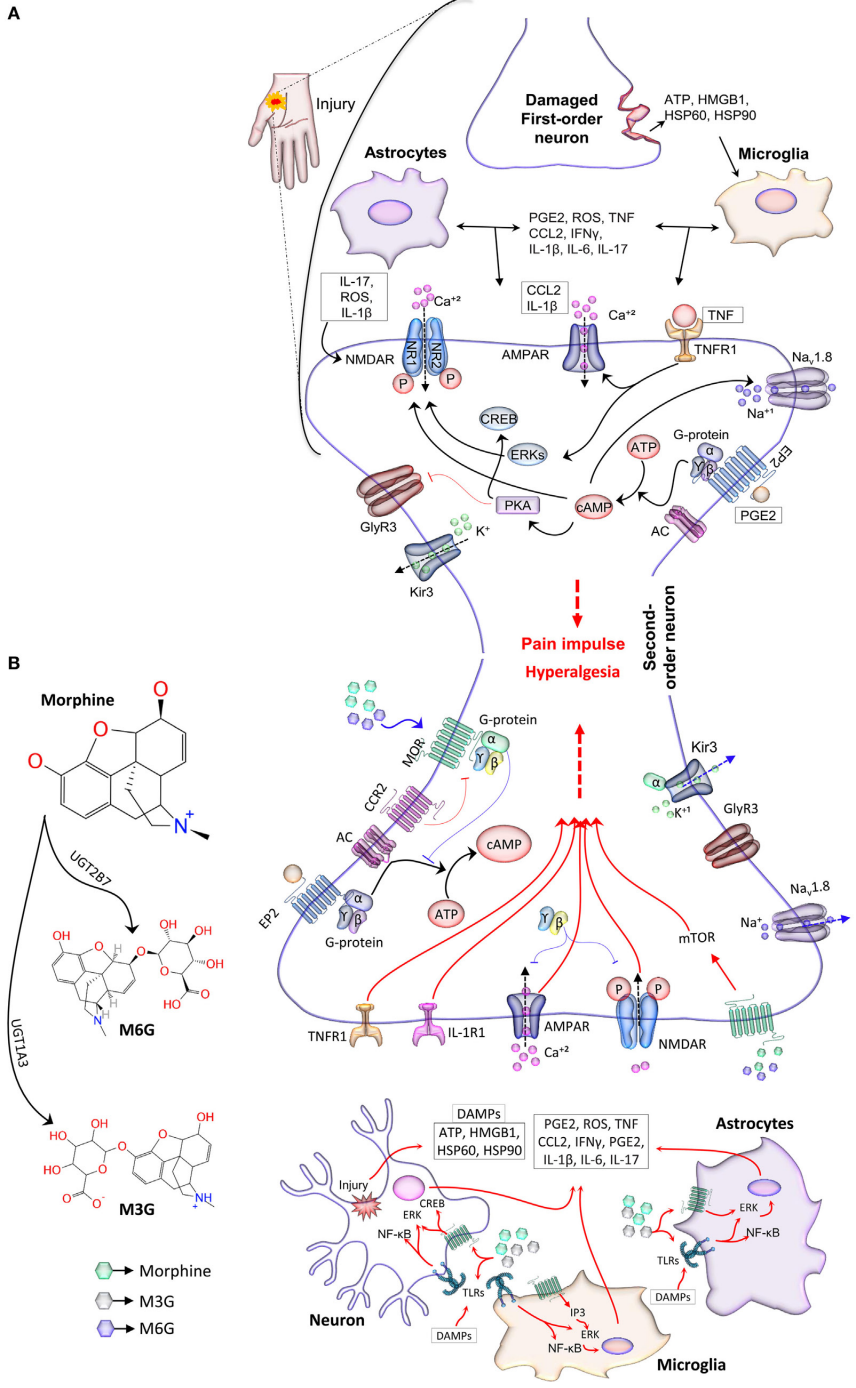
The neuroimmune interface and the role of glia in the pain response. **(A)** Reactive immunocompetent cells located at the site of injury release soluble mediators that diffuse into the neuroimmune interface and bind to synaptic terminals. These mediators modulate excitatory and inhibitory synaptic transmission and lead to nociceptive hypersensitivity. Mediators such as CCL2 and IL-1β elevate TNFR1 and AMPA signaling and the expression of Ca^2+^-permeable AMPARs. TNF also increases the phosphorylation of ERKs through the TNFR1 pathway, which then activates NMDAR activity. ROS and IL-17 induce the phosphorylation of the NR1 subunit of NMDAR. Other subunits, NR1, NR2A, and NR2B, are phosphorylated by IL-1β. Together, these mediators increase the influx of Ca^2+^ ions, thereby augmenting pain sensation. PGE2 activates AC through receptor stimulation of the G-protein (G), which then catalyzes the conversion of ATP into cAMP. The G-dependent rise in cAMP level is crucial for neuronal excitability. cAMP regulates the phosphorylation of ion channels, Na_v_1.8 and NMDAR, through PKA (11). PGE2-dependent EP2 signaling also leads to the PKA-dependent inhibition of glycinergic neurotransmission *via* GlyR3 receptors. Taken together, mediators released by glia, injured neurons, or other central immune cells promote pain sensation and result in pathological pain in severe conditions. **(B)** Opioid-induced analgesia and hyperalgesia. Morph is glucuronidated into M3G and M6G in hepatocytes. Morph and its MOR active metabolite, M6G, produce analgesic effects by modulating the Ca^2+^ and K^+^ ion channels through MOR mediated signal transduction (blue arrows). MORs are associated with G-proteins; after dissociation, the G_α_ subunit of the G-protein moves and directly interacts with the G-protein-gated inwardly rectifying K^+^ channel, Kir3 (12–14). The dissociated G_α_ subunit also decreases synaptic transmission partly by inhibiting ACs, thereby reducing the cAMP-dependent Ca^2+^ influx (15). Channel deactivation occurs after hydrolysis of GTP to GDP and G_α_ removal from the channel. This process causes cellular hyperpolarization and inhibits tonic neural activity. Opioid receptor-induced inhibition of Ca^2+^ conductance is mediated by binding of the dissociated G_βϒ_ subunit directly to the channel. This binding event is thought to reduce voltage activation of the channel pore opening and to enhance the analgesic effect of opioids. TLRs, expressed on neurons, glia, and other neuroimmune cells, are activated non-stereoselectively by both active and inactive isomers of Morph and its opioid-inactive metabolite M3G (8). Microglial activation and subsequent proinflammatory cytokine release sensitize neurons and diminish the analgesic effects of Morph and M6G (8, 16–18). This mechanism is thought to explain the negative effects of opioids (red arrows). Abbreviations: AC, adenylyl cyclase; AMPA, α-amino-3-hydroxy-5-methyl-4-isoxazole propionic acid; AMPAR, α-amino-3-hydroxy-5-methyl-4-isoxazole propionic acid receptors; ATP, adenosine triphosphate; cAMP, cyclic adenosine monophosphate; CCL2, CC-chemokine ligand 2; CREB, cAMP response element-binding protein; EP2, prostaglandin E receptor 2; ERK, extracellular signal-regulated kinase; GlyR3, glycine receptor 3; IFN, interferon; IL, interleukin; IP3, inositol triphosphate; Morph, morphine; MOR: μ-opioid receptor; mTOR, mammalian target of rapamycin; M3G, morphine-3-glucuronide; M6G, morphine-6-glucuronide; NMDAR, *N*-methyl-d-aspartic acid receptors; PGE2, prostaglandin E2; PKA, protein kinase A; HSP, heat shock protein; HMGB1, high mobility group box 1 protein; ROS, reactive oxygen species; TNF, tumor necrosis factor; TNFRs, tumor necrosis factor receptors.

Microglial cells cover approximately 15% of all cells in the CNS and are primarily derived from primitive myeloid precursors (9). Microglial cells play a crucial role in the tetrapartite synapse, relay post-injury plastic changes, and induce central sensitization. During neuronal injury or other pathological conditions, activated microglial cells release proinflammatory mediators that activate nearby glial and neuronal cells in the tetrapartite synapse. This potentiates the neuroinflammatory response, which can also lead to hyperalgesia (7, 10). The role of glia in normal pain progression is illustrated in Figure 1.

Astrocytes also play a key role in neuroimmune signaling; they are involved in the modulation of glutamate uptake by altering glutamate transporters and releasing proinflammatory cytokines that heighten pain sensation (19–21). On activation of astrocytes by neuronal injury or activated microglial cells, the expression of plasma membrane-localized glutamate (glutamate transporter 1) and glutamate-aspartate transporters is downregulated in the astrocytes, thereby resulting in decreased glutamate uptake and thus increased nociception (22). Moreover, these cells recognize DAMPs and induce the receptor-dependent activation of cellular kinases, c-jun N-terminal kinase (JNK), and extracellular signal-regulated kinase, leading to the release of proinflammatory cytokines (10) and thus pathological and inflammatory pain progression (Figure 1A).

## Molecular Mechanism of Pain Sensation and Progression

Injury caused by physical or chemical stressors leads to high-threshold biochemical activity and release of elevated inflammatory mediators from damaged neurons, which initiate neuroimmune signaling at the innervation level (23). Astrocytes and T cells are reported to receive and convey neurochemical signals; however, microglial cells initiate the response to the mediators released by damaged neurons. These mediators include chemokines, adenosine triphosphate, and DAMPs, in particular high mobility group box 1 protein, heat shock protein (HSP) 60, and HSP90 (2, 24). A transition into reactive gliosis takes place when microglia detect these signals. Reactive gliosis leads to astrogliosis and the infiltration of peripheral immune cells owing to the release of chemokines, cytokines, and DAMPs (Figure 1A). Beside other cytokines and chemokine-recognizing receptors, recent studies have found that glia also express TLRs, which respond to the DAMPs released by damaged neurons or other central immune cells (25, 26). Heightened activation of TLR-expressing glial cells has been reported to play a crucial role in neuropathic pain. The finding that TLRs play a critical role in the nervous system-related pathologies and are capable of interacting with ligands other than those associated with pathogens has further emphasized that TLR-inhibiting molecules could be useful in alleviating glial-mediated allodynia (27).

## The Role of Glial TLRs in Pain

**Figure 2:**
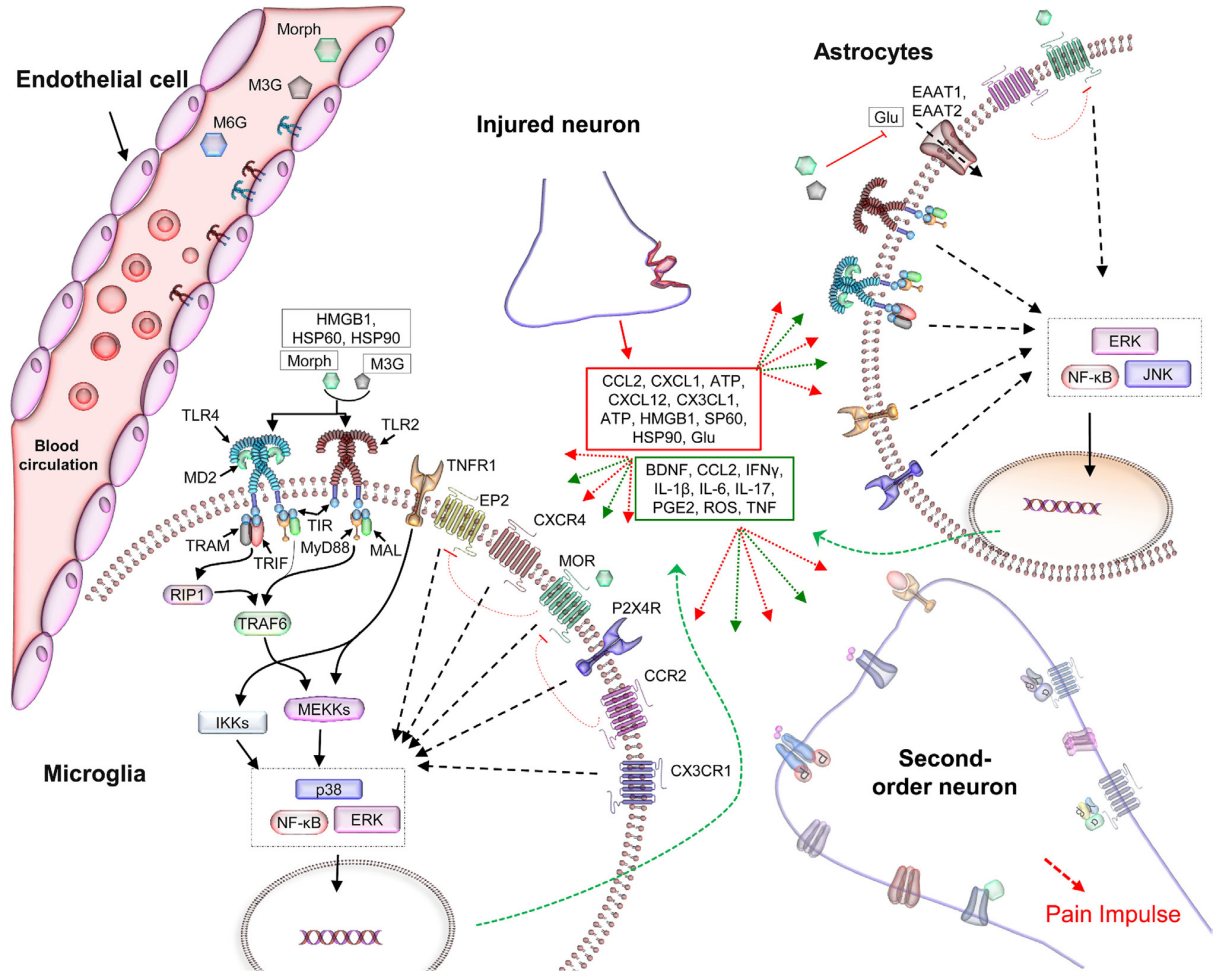
The role of TLRs in hyperalgesia and the negative effects of opioids in analgesia. Soluble mediators, released by injured tissues and immune cells in the neuroimmune interface, interact with the synaptic terminals and TLRs on glial and endothelial cells (25, 26). This activates complex signaling processes that intersect at multiple points, modulating inhibitory and excitatory synaptic processes and thereby resulting in nociceptive hypersensitivity. Among the TLRs, TLR2 and TLR4 have been widely reported to be associated with gliosis in studies of the neuroimmune interface (2, 31, 32). Microglia, astrocytes, and endothelial cells, located in the CNS, express TLR4 (32) and TLR2 (17), which can recognize potentially harmful substances such as DAMPs released by the damaged tissues and neurons (red box) and activate an immune-like response (18, 33). This immune cascade activates second-order neurons that transmit the pain impulse to the CNS (Figure 1). Activation of TLRs in CNS cells provokes immune-like responses *via* the production of proinflammatory cytokines (green box). These cytokines are then released into the extracellular environment, where they activate other receptors within the synaptic cleft (red and green dotted arrows) (34). Together, these activated receptors exacerbate the inflammatory response, leading to allodynia (35). In addition, the signaling complex modulates ion channels and the electrical potential of the CNS, and reduces the analgesic effect of morphine and its opioid receptors’ active metabolite, M6G. Abbreviations: ATP, adenosine triphosphate; BNDF, brain-derived neurotrophic factor; CCL2, CC-chemokine ligand 2; DAMPs, danger-associated molecular patterns; EP2, prostaglandin E receptor 2; ERK, extracellular signal-regulated kinase; HSP, heat shock protein; IFNγ, interferon γ; IKK, inhibitor of κB-kinase; IL-1β, interleukin-1β; JNK, c-jun N-terminal kinase; MAL, MyD88-adapter-like; MyD88, myeloid differentiation protein 88; Morph, morphine; MOR, μ-opioid receptor; M6G, morphine-6-glucuronide; NF-κB, nuclear factor κ-light-chain-enhancer of activated B cells; PGE2, prostaglandin E2; RIP1, receptor-interacting protein-1; TAB, TAK1-binding protein; TIR, toll/IL-1 receptor homology domain; TNF, tumor necrosis factor; TRAF6, TNFR-associated factor 6; TLRs, toll-like receptors; TRAM, TRIF-related adaptor molecule; TRIF, TIR-domain-containing adapter-inducing interferon-β.

The association between pain and TLRs can be traced back to the era before the discovery of lipopolysaccharides as the ligand of TLR4. TLRs are receptors expressed on various types of cells, including those present in the CNS and constitute a vital link between the immune system and the CNS. In addition to their expression on immunocompetent cells, TLRs have been reported to be expressed on endothelial and neuronal cells that are originally considered non-immunocompetent cells (25). TLRs expressed on neuroimmune cells have mainly been linked to hyperalgesia; however, accrued evidences now confirm that glial TLRs are also involved in suppressing inflammation and neuronal repair (7, 28). The role of TLRs in pathological pain has been well documented in the literature (8, 18, 25). TLRs expressed by central immune cells are mainly activated by DAMPs, released from damaged neurons at the site of injury, which further enhance the proinflammatory cascade and leads to the augmentation of the pain impulse ultimately resulting in pathological pain (29, 30). The involvement of TLRs in nociception makes them a critical component of analgesics and other pain-relieving substances (Figure 2).

The involvement of TLRs in pathological pain has recently been highlighted in studies on animal models, wherein TLR4 is determined as a pain initiator (32, 36). Furthermore, the inhibition of the TLR2 and TLR4 pathways has also been reported to prevent and relieve neuropathic pain in animal models (37, 38). In addition to the well-studied role of TLR4 in neuropathic pain, other TLRs, such as TLR2 and TLR3, have also been reported to be involved in the potentiation of pain in preclinical pain models (39–41). Kim et al. suggested that glial cells, activated by neuronal injury, participate in processes leading to pain hypersensitivity through the direct activation of TLR2; this was further confirmed in TLR2-knockout mice, wherein the induced allodynia vanished (42). The association between TLRs, glia, and neuropathic pain has been extensively investigated *in vitro*. The discovery that glia and some neurons in the CNS and PNS express TLRs has highlighted their substantial role in the modulation of neuropathic and inflammatory pain. It should be noted that, while resolving the problem of hyperalgesia, using analgesics that can activate both μ-opioid receptor (MOR) and TLRs would worsen the general scenario; alternatively, pain killers, such as psychotropic agents, including ketamine and clonidine, COX-inhibitors, and non-steroidal anti-inflammatory drugs should therefore be used for pain relief.

## Opioid-Induced TLR-Dependent Hyperalgesia

Opioids, considered the benchmark therapy for both chronic and acute pain, are also associated with paradoxical hyperplasia. Even though opioids are continuously used in relieving pain, their immune signaling mechanisms in the CNS are not well documented. It has been reported that, apart from their direct interaction with opioid-receptors expressed on glial and neuronal cells, opioids can interact with other receptors and activate exacerbated immune-like signaling in the CNS (8). Neuroinflammatory cells, including glia, have been associated with opioid-induced hyperalgesia, and this association has been well studied over the past decade (1).

Generally, opioids including morphine produce analgesic effects by modulating Ca^2+^ and K^+^ ion channels through MOR-mediated signal transduction. MORs are associated with G-proteins; after dissociation, the G_α_ subunit moves and directly interacts with the G-protein-gated inwardly rectifying K^+^ channel, Kir3 (12–14). The dissociated G_α_ subunit also decreases synaptic transmission partly by inhibiting adenylyl cyclase, thereby reducing the cyclic adenosine monophosphate-dependent Ca^2+^ influx (15). Channel deactivation occurs after hydrolysis of GTP to GDP and G_α_ removal from the channel. This process causes cellular hyperpolarization and inhibits tonic neural activity. Opioid receptor-induced inhibition of Ca^2+^ conductance is mediated by binding of the dissociated G_βϒ_ subunit directly to the channel. This binding event is thought to reduce voltage activation of the channel pore opening and enhance the analgesic effect of opioids (Figure 1B).

The off-target exacerbated signaling at the neuroimmune interface and the classical analgesic mechanism have been linked to the stereoselective interaction of morphine and its metabolites, M3G and morphine-6-glucuronide (M6G), with MOR and TLRs. Morphine, when administered into the body, is glucuronidated into M3G and M6G in the hepatocytes. M6G stereoselectively binds to the MOR (43, 44), while morphine, with and without MOR activity, and M3G have been reported to oppose analgesia and boost nociception (8, 45, 46). Morphine, a classical opioid, has been shown to bind non-stereoselectively to TLR4/MD2 and to activate the TLR4 pathway (47, 48). Besides morphine and its opioid-inactive metabolite, M3G, other clinically significant opioids have also been reported to bind to the TLR4/MD2 complex (1, 47, 49). After binding to the TLRs expressed on glia and dorsal root ganglion neuron, opioids, including morphine and M3G, activate the downstream signaling pathways. Activation of TLRs in these cells has been linked to the release of proinflammatory mediators, including but not limited to nitric oxide, reactive oxygen species, interleukins, interferons, monocyte chemotactic protein-1, CC-chemokine ligand 5, CC-chemokine ligand 2, CXCL10, inducible nitric oxide synthase, and prostaglandin E2. Excluding TLR3, all TLRs are known to convey their signaling *via* myeloid differentiation protein 88 (MyD88). After activation, MyD88 initiates a signaling cascade, which ultimately results in the release of proinflammatory mediators. These mediators exacerbate the pain sensation and progression leading to nociception and hyperalgesia. The molecular mechanism of opioid-induced TLR-dependent hyperalgesia has been illustrated in Figure 2. Ligands that can selectively block the signaling of TLRs have therefore been used to restore the analgesic effect of opioids in experimental animal models and *in vitro* experiments. Accordingly, the involvement of TLRs in the pharmacodynamics of opioids has become obvious.

## Conclusion

After understanding the role of individual cells at the tetrapartite synapse and the neuroimmune interface in general, one could suggest that pain initiated by a stimulus or pathologically, is the result of a complex neuroimmune-signaling cascade. Furthermore, TLRs expressed on the cells present in the nervous system are pivotal in the maintenance and sustention of neuropathic pain and counter the analgesic effect of opioids. To avoid negative side effects of opioids, the development of non-opioid therapies for nociceptive pain needs serious consideration; however, some alternative therapies are already being investigated in preclinical trials. This will not only resolve the problem of heightened pain sensitivity in pathological conditions but also reduce the chances of opioid-related drug abuse in patients with depression, stress, or acquired immunodeficiency syndrome.

## Author Contributions

MS and SC designed the study, wrote the manuscript, proofread, and approved this work. MS generated the figures and SC approved them.

## Conflict of Interest Statement

The authors declare that the research was conducted in the absence of any commercial or financial relationships that could be construed as a potential conflict of interest.
